# Advancements in Neighboring-Based Energy-Efficient Routing Protocol (NBEER) for Underwater Wireless Sensor Networks

**DOI:** 10.3390/s23136025

**Published:** 2023-06-29

**Authors:** Sayyed Mudassar Shah, Zhaoyun Sun, Khalid Zaman, Altaf Hussain, Inam Ullah, Yazeed Yasin Ghadi, Muhammad Abbas Khan, Rashid Nasimov

**Affiliations:** 1 Information Engineering School, Chang’an University, Xi’an 710061, China; 2 School of Computer Science & Technology, Chongqing University of Posts and Telecommunications, Chongqing 400065, China; 3 Department of Computer Engineering, Gachon University, Seongnam 13120, Sujeong-gu, Republic of Korea; 4 Department of Computer Science, Al Ain University, Abu Dhabi P.O. Box 112612, United Arab Emirates; 5 Department of Electrical Engineering, Balochistan University of Information Technology, Engineering and Management Sciences, Quetta 87300, Pakistan; 6 Department of Artificial Intelligence, Tashkent State University of Economics, Tashkent 100066, Uzbekistan

**Keywords:** neighbor head node selection, Co-UWSN, CEER, NBEER, autonomous underwater vehicles

## Abstract

Underwater wireless sensor networks (UWSNs) have gained prominence in wireless sensor technology, featuring resource-limited sensor nodes deployed in challenging underwater environments. To address challenges like power consumption, network lifetime, node deployment, topology, and propagation delays, cooperative transmission protocols like co-operative (Co-UWSN) and co-operative energy-efficient routing (CEER) have been proposed. These protocols utilize broadcast capabilities and neighbor head node (NHN) selection for cooperative routing. This research introduces NBEER, a novel neighbor-based energy-efficient routing protocol tailored for UWSNs. NBEER aims to surpass the limitations of Co-UWSN and CEER by optimizing NHNS and cooperative mechanisms to achieve load balancing and enhance network performance. Through comprehensive MATLAB simulations, we evaluated NBEER against Co-UWSN and CEER, demonstrating its superior performance across various metrics. NBEER significantly maximizes end-to-end delay, reduces energy consumption, improves packet delivery ratio, extends network lifetime, and enhances total received packets analysis compared to the existing protocols.

## 1. Introduction

Underwater wireless sensor networks (UWSNs) occupy a pivotal role in the exploration and examination of underwater environments. UWSNs have the potential to gather valuable data from regions submerged under water, which make up over 70% of Earth’s surface [1,2]. These networks consist of numerous autonomous underwater vehicles (AUVs) that gather information from deployed sensor nodes [3]. Major challenges in underwater communication include node failures, limited bandwidth, and extended propagation delays. Although marine life research has obtained significant interest in recent times, there has been a growing focus on this area due to heightened public awareness. The oceans cover approximately two-thirds of the Earth’s surface, and their vitality is essential for human survival.

**Figure 1:**
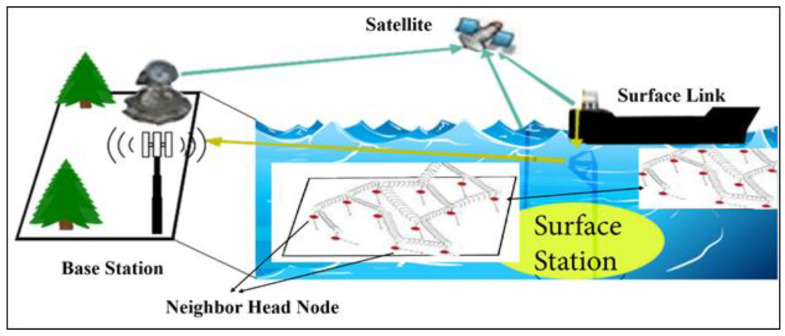
Framework for neighbor-based energy-efficient routing protocol.

In addition to providing essential resources for the global economy, the oceans absorb a significant amount of carbon dioxide emissions, thereby regulating the temperature of the planet. Despite the significance of oceans, an astounding 94% of their potential remains unexplored, according to estimates. In this context, UWSNs have the potential to effect significant change. It has been demonstrated that UWSNs are an effective alternative to traditional wired and non-communicative technologies for monitoring and investigating underwater environments. The underwater wireless communication capabilities of UWSN nodes enable monitoring, reconfiguration of operating systems, and fault detection [4]. This innovative technology aids researchers and industry professionals in underwater surveillance. Ocean exploration, marine and offshore sampling, navigational aids, and military security cameras are among the many applications of sonar. UWSNs rely on water-based communication [5]. While radio waves function well in terrestrial wired sensor networks (TWSNs), they are significantly attenuated in aquatic environments. It is essential to observe that sound waves in water have a very narrow bandwidth and travel six orders of magnitude slower than radio waves in the air. A scenario of NBEER routing protocol is illustrated in Figure 1.

In addition, underwater acoustic modems consume significantly more power than radio modems. Multipath effects, Doppler frequency shift phenomena, and other disruptions contribute to transient connection failures [6]. Additionally, most applications involve nodes that move with water flow. GPS systems do not function underwater [7]. This analysis concludes that routing protocols designed for TWSNs are inappropriate for underwater applications. UWSNs comprise a diverse range of sensors and vehicles positioned strategically within a specific region to engage in collaborative efforts for observation duties. Although practical applications and implementations of underwater transportation have existed for quite some time [8,9], a considerable number of individuals still possess limited familiarity with underwater networks. The identification of subsea assets and hydrocarbon fields is critical. Additionally, the mapping of underwater cable installation routes is essential. UWSNs are also capable of detecting multiple threats, such as tsunamis. Facilitating submerged transportation is essential for the accomplishment of these objectives. Wireless underwater acoustic networks make these applications possible. UWSNs have a variety of applications, including the detection of underwater hydrocarbon fields and natural disasters and the determination of submarine positioning routes [10,11].

The deployment and utilization of underwater sensors, data acquisition through observational activities, and equipment retrieval constitute integral aspects of underwater operations. However, this approach suffers from several drawbacks, notably the absence of real-time monitoring, the inability to configure operating systems, the absence of fault detection, extended propagation delays, and limited bandwidth availability [12,13]. In UWSNs, energy efficiency remains among the myriad of complexities encountered, and addressing this facet proves to be a formidable endeavor. Numerous researchers have investigated the most efficient ways to reduce the energy consumption of nodes. Cooperative underwater wireless sensor network co-operative (Co-UWSN) and Co-operative energy-efficient routing (CEER) protocols are two of these methods. Co-UWSNs and CEER are selected because they can enhance network performance while also serving as potential destinations and relays. However, the disadvantage of Co-UWSN and CEER is that collaboration occurs at the node level, which increases energy consumption and sensor node separation [14].

The primary contributions of this study include the following:

- Energy consumption and latency reduction: The proposed routing technique introduces a novel routing scheme aimed at minimizing energy consumption and addressing latency issues. This is achieved through the utilization of neighbor node data cooperation, enabling efficient data transfer to the base station.
- Neighbor node cooperation: The proposed method incorporates neighbor node cooperation to optimize energy consumption. By selecting neighbor nodes with high residual energy as neighbor head nodes (NHNs), data can be efficiently transmitted to the sink node located on the water surface.
- Enhancing data reliability: Recognizing that direct data transmission does not guarantee reliability, the proposed approach employs neighbor node cooperative data forwarding. This technique effectively reduces end-to-end delay and enhances the network’s overall lifetime.
- Performance evaluation: The research evaluates the performance of the routing scheme using the MATLAB simulation tool. The results demonstrate improvements in energy consumption, end-to-end delay, packet delivery ratio (PDR), transmission loss, and the number of packets received.

This article is structured as follows. The first section provides a concise introduction to the research topic, the research questions or objectives, and context for the study. In Section 2, a literature review is presented. Section 3 provides a thorough explanation of the research methodology. In Section 4, the simulation results are discussed. Section 5 provides a comprehensive conclusion.

## 2. Literature Review

The authors investigated various parameters that have an impact on energy-efficient routing in UWSNs. We analyze the factors that influence the selection of optimal routing paths in order to minimize energy consumption and prolong the network lifetime. The examined parameters include node density, transmission range, routing protocols, and energy-aware algorithms. By understanding these key elements, we can develop effective strategies to enhance the energy efficiency of UWSNs, leading to improved network performance and extended operational duration nodes for the connection. The relay node transmits the packet as quickly as possible to the destination node, which accepts and acknowledges the packet. To receive or rebroadcast packets efficiently, time-reliable scheduling must be established between the relay, source, and destination nodes [15]. This resolves the issue of packet loss. According to simulation case studies, the proposed approach outperforms some existing schemes in terms of packet delivery to the destination [16] A micro-ANP protocol architecture was devised for UAN. It consists of three layers: application, network transport, and physical. In addition, this paper provides a comprehensive description of a handshake-free consistent communication method based on the micro-ANP design and recursive LT encoding.

Diverse structures of hydroacoustic communication networks are investigated in [17,18,19,20], considering their potential for UWSN consumption reduction. Multipath fading, rapid fading of acoustic signals, and extended propagation delays are characteristics of these structures. The absolute selection of forwarding nodes frequently results in node demise, resulting in unequal energy conservation and network vacancies. The objective of their model, which is known as the geographic opportunity routing model, is to reduce energy tolerance deficits and imbalances. It is a mobile-assisted model that prevents UWSN interference. Among other advantages, the network volume is divided into small logical units to minimize interference and provide a more informed routing consideration for energy-efficient protocol consumption.

The authors of [21] suggested assigning an optimal number of transponder nodes to each cube based on its proximity to the destination to prevent invalid events within the cubes. In addition, removable receivers are used to improve packets from the vacant section, thereby reducing data traffic on intermediate nodes. To validate their proposed work, extensive simulations were conducted with a focus on exploiting and maximizing the network’s lifecycle and packet delivery rate. According to the authors, the problem of determining the AUV’s route must be addressed in order to enhance the value of information (VoI) of the actual data transmitted to the sea sink node. They proposed an adaptive and greedy AUV routing algorithm that directs AUVs to collect data from nodes whose proper operation depends on VoI and their data.

The authors presented a framework for integer linear programming that precisely models the estimated setup to generate a route for the AUV to collect and transmit more VoI data. This allowed them to evaluate the effectiveness of the AUV’s routing strategy. Experiments revealed that greedy adaptive AUV path planning (GAAP) consistently provided more than 81% of the optimal centralized VoI solution based on the integer linear programming (ILP) model. The cases examined led to the development of a route that enabled the autonomous underwater vehicle (AUV) to be deployed for data collection. AUVs collect and transmit data with an extraordinarily high V-o-I while adhering to operational constraints. They compared the efficacy of GAAP and other methods for transferring AUVs between sensing nodes, including random routes, traveling salesman problem (TSP)-based routes, and “lawnmower” patterns. According to their findings, GAAP consistently outperforms other schemes in terms of transmitting V-o-I while also attaining greater power efficiency [22,23,24,25,26].

The authors discussed a variety of WSN techniques, including energy-aware routing, as well as the parameters influencing energy-conscious routing in WSNs. In addition to demonstrating algebraic and graphical modeling of these factors, this knowledge enables students to design such algorithms more efficiently, evaluate the practicality of existing algorithms, and extend them. This section explores the various techniques employed using available routing algorithms to resolve these issues and become more energy-aware. In addition, the researchers’ suggestions for development are taken into account.

The authors of [27,28,29,30,31,32] introduced a novel communication methodology known as context-aware communication (CACA-UAN). This mechanism is a scheme for addressing underwater networks, with the goal of improving the overall efficacy of objects in underwater networks through the application of artificial intelligence. After concluding their research, they determined that the CACA-UAN system increases the dependability and effectiveness of underwater communication schemes.

The authors proposed an energy-aware and avoidable invalid routing protocol (EAVARP) as a novel routing approach based on hierarchical and data acquisition phases that complements existing routing methods [33,34]. They argued that designing and planning routing protocols with extended network lifetimes and higher packet delivery rates in complex underwater environments presents significant challenges for UWSNs [35,36]. As mentioned previously, the proposed protocol is hierarchical, with several underwater concentric shells formed around the sink nodes and the sensor network’s nodes separated into different shells during the hierarchical portion of the protocol [37,38]. Sink nodes are assigned tasks based on time intervals to ensure the network’s real-time and temporal validity, thereby allowing the network’s physical shape to be measured [39,40,41,42,43]. A detail evaluation of the literature review has been given in Table 1.

## 3. Methodology

The deployment phase of the nodes is crucial for their success. During this phase, 250 nodes are randomly deployed. The nodes are divided using three tiers and three nearest-neighbor schemes (NNSs), with three neighbor head nodes per tier and nine NHNs in total. Twelve receivers(sink) are placed on the water surface, and one base station is positioned offshore. All NHNs in the upper layer (layer 3) gather data from the nearest sensor nodes and transmit them directly to the sink. The sink sends the data to the base station. The rest of the procedure follows the same process for data transmission from nodes to the best station. For a better understanding of the proposed NBEER, the research methodology is described in the below steps.

**Figure 2:**
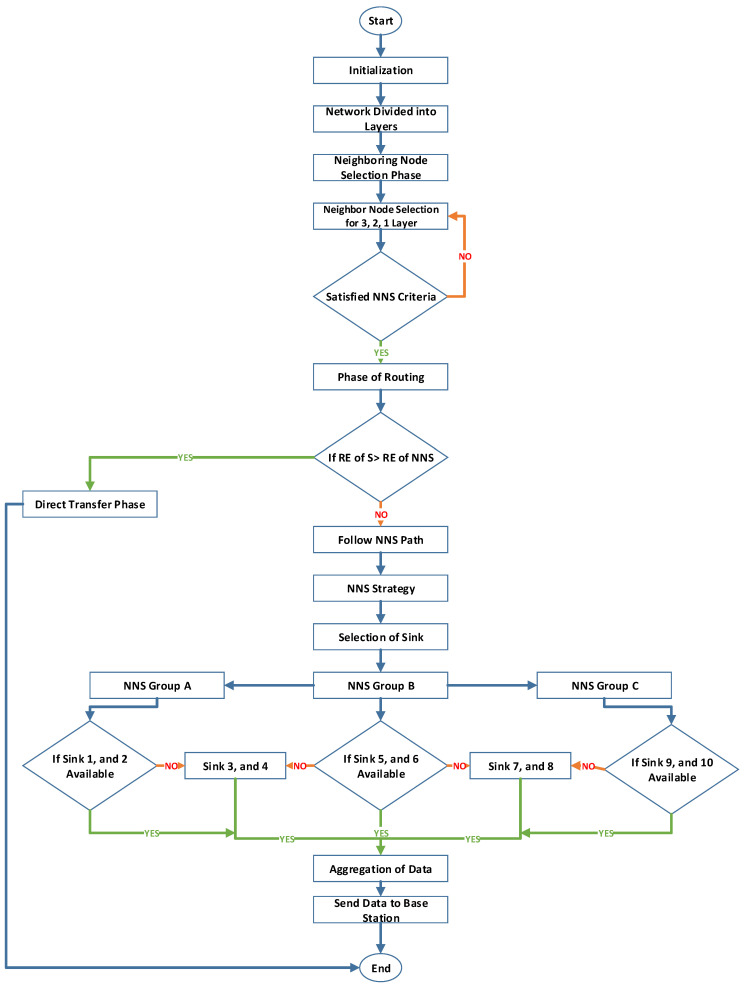
Flowchart for NBEER protocol.

The proposed work purely focused on neighbor node identification (NNI) and NNSs as shown in Figure 2. The experiment was carried out by placing different random clusters and moving nodes underwater. Each node has the ability to detect, identify, and forward the routing path to the nearest one. This method is carried out by the route discovery and route maintenance. The flooding mechanism takes place here, which uses this idea to discover the nearest node.

### 3.1. System Model

The system model is the mathematical implementation and illustration of the proposed work. Here, each step and process has been carefully considered and implemented, taking into consideration the proposed major concern, which is NSNSs and the selection of the neighbor nodes. The first step is illustrated as the deployment phase of nodes, in which different nodes are deployed as mentioned. These nodes are deployed for testing and identification as well as the discovery of nearby nodes. The performance is evaluated too with the other traditional protocols of UWSNs. The clusters are also selected arbitrarily and randomly because of the moving nodes in the scenario of UWSNs. These clusters can be adaptive in the proposed process.

Using a mathematical formulation, the NBEER protocol for UWSNs is designed. By incorporating an NHNS mechanism and collaboration techniques, this routing protocol is developed specifically for UWSNs. Following are the various steps and techniques used, explained in detail.

#### 3.1.1. Node Deployment

This phase denotes the overall range and procedure of the node’s deployment for the proposed protocol. For testing analysis and purpose, the range of the proposed nodes is introduced. With different random clusters, these nodes are deployed underwater. A total of 250 nodes are deployed underwater in three layers, with each layer consisting of three clusters. This results in a total of nine (9) clusters. Twelve sink nodes are deployed on the ocean’s surface to collect data from the underwater nodes. One base station is installed on the ground near the surface of the ocean.

#### 3.1.2. Data Processing

This stage denotes the processing of the data, including how the actual data will be processed and originated from the sender and how they will be successfully delivered to the destination without any loss, packet loss, delay, or consumption of energy. This means that the protocol will have to consume less energy. The base station processes the aggregated data for further analysis and application. The NBEER protocol uses this system paradigm to increase data transmission efficiency and reliability in UWSNs while consuming less energy and extending network lifetime.

#### 3.1.3. Node Discovery

This stage denotes the discovery of the nodes. Nearby nodes will be discovered using the nearby node selection process. So, the NNS procedure is applied here for discovery and identification. Each node is made aware of the presence and location of adjacent nodes, as well as sink nodes deployed in the water. This step ensures that each node is conscious of its neighbors and potential communication paths.

#### 3.1.4. Route Analysis

Route analysis is performed to effectively analyze the data and to check the performance and integration of the data. Also, route discovery and analysis take place during this step. To ensure efficient data transmission, all potential routes to the sinks are examined and analyzed exhaustively. This analysis optimizes the routing procedure and reduces energy consumption.

#### 3.1.5. Depth Communication

The depth readings are transmitted by the sensors to adjacent nodes and receivers. Each node transmits packets with information including node ID, energy status, and depth. The receiver exchanges welcome packets with the nodes to obtain information about each node.

#### 3.1.6. Neighboring Node Identification (NNI)

The core method of NNI is given in Equations (1)–(3), respectively. Now, each equation has its unique characteristics. The discovery of nodes takes place in this step, but the nodes must find the best nodes among all the nodes, that is, the nodes with the highest energy level, and the highest and most stable communication path.

Each node transmits welcome packets to identify other nodes in its transmission range. The nodes maintain the isolation line between contiguous nodes below the depth threshold, which enables the identification of the most appropriate and optimal transponder nodes for data transmission. By identifying neighboring nodes, analyzing routing paths, and exchanging depth information, the neighboring node identification lays the groundwork for efficient communication in UWSNs. This procedure ensures that subsequent phases of data transmission and routing are both efficient and effective.

(1) $$W_{g}=\frac{min({PL}_{SiRi},{PL}_{Ri,Di})+min(({SNR}_{Si,Ri},{SNR}_{RiDi})}{max(({R.E}_{Ri},{R.E}_{Di})}$$

(2) $$PL=20\;n\;{Log}_{10}\left(d\right)+c$$

(3) $$SNR=\frac{P_{signal}}{P_{Power}},{SNR}_{db}=10{log}_{10}\left(SNR\right)$$

In the equations, PL represents the path loss between the communicating nodes (*Si*, *Ri*, and *Di*). *Si* stands for the sender node, *Ri* for the relay node, and *Di* for the destination/receiver node. The signal-to-noise ratio (SNR) quantifies the quality of the communication link between two terminals. For the links between the source and the receiver nodes and between the sender and destination nodes, the SNR values (SNR (*Si*, *Ri*) and SNR (*Ri*, *Di*)) are calculated. Renewable energy, also known as residual energy, is the energy remaining in the nodes after several cycles of communication. This is an essential consideration when selecting NHNs, as it ensures that only nodes with sufficient energy are chosen to anticipate in the communication procedure, thereby extending the network’s lifetime. The weighted gain (*W*, *g*) identifies the most efficient NHN (*Ri*) for data transmission among the sender (*Si*) and receiver (*Di*) nodes by considering path loss, SNR, and residual energy. This strategy maximizes the overall performance of the submerged wireless sensor network via ensuring that the transmission process is both energy-efficient and reliable.

#### 3.1.7. Choosing the Neighbor Head Node

When the power level surpasses a specific threshold, an individualized message, known as a “welcome” message, is dispatched to every neighbor head node (NHN) situated within the network domain. By leveraging the strength of the incoming signal, each node adeptly computes its relative distance from the receiver, thus facilitating the formation of tire combinations of varying sizes [18,19]. Upon reception of this message, the node promptly evaluates its own competition radius, encompassing neighboring nodes, and subsequently generates a comprehensive report to be relayed to the sink. This report encapsulates crucial information, including the node’s current expected remaining power, the corrected nearest-neighbor (NN) value, and the unique node identifier.

In the context of our proposed NBEER methodology, a node’s eligibility to assume the role of an NN is predicated solely upon its possession of greater residual power in comparison to the nodes within its radius or neighbor. In order to engender a heterogeneous composition of clusters, it is imperative that each node undergoes a meticulous process to identify its NHN. Remarkably, nodes endowed with more remaining power within the NBEER framework shoulder more vital responsibilities, imparting a distinct character to the ensuing cluster configuration. Consequently, clusters positioned farther from the sink, which boast heightened residual power, adopt a single-hop topology, and accommodate a greater number of nodes relative to clusters in closer proximity to the sink, which instead employ a multi-hop topology. As the spatial span between sensor nodes and sinks widens, sensor nodes blessed with augmented residual energy play a pivotal role in replenishing their respective neural networks.

Moreover, it is imperative to exercise caution with nodes possessing diminished remaining power, as their assigned NN values should be reduced to mitigate the risk of premature depletion. The evaluation and representation of the NN parameter constitute indispensable facets within the system’s operation and optimization.

(4) $$NN=\left[\frac{1-W(X_{Max}-X(Qa-Bs))}{X_{Max}-X_{Min}}-G\left(1-\frac{I_{remaning}}{I_{maximum}}\right)\right]{NN}^{0}$$

In the proposed system, the variable *X*($Q_{a}$,*BS*) denotes the distance between $Q_{a}$, i.e., a sensor node and the base station (BS). The weights w and g are factors used in the calculations. The value ${NN}^{0}$ represents the maximum number of nearest neighbors, NN, considered. The variables $I_{remaning}$ and $I_{max}$ represent the remaining power of a node and the maximum energy of the main node, respectively. It is assumed that all nodes possess equal energy levels; $X_{max}$ and $X_{min}$ indicate the maximum and minimum distances between the base sink and the sensor nodes.

Upon receiving a push notification from a sensor, the system proceeds to select the neighboring master node from the closest nodes. Subsequently, the base station generates a matrix and broadcasts the notification to all sensor nodes within the network. This matrix is then made visible for reference and analysis.

Overall, the system incorporates distance calculations, neighbor selection, and matrix broadcasting to facilitate efficient communication and coordination among the sensor nodes and the base station.

In our wireless sensor network, let us delve into the intriguing realm of variables and their fascinating roles. Behold, the mystical symbol *X*($Q_{a},BS)$ representing the ethereal distance $Q_{a}$ between a sensor node and the esteemed base station (BS). As we journey deeper, we encounter w and g, enigmatic factor weights, and witness the emergence of ${NN}^{0}$, a symbol denoting the pinnacle of NN’s existence. Amidst this symphony of knowledge, we encounter the remaining I, a measure of a node’s unwavering power, while $I_{max}$ shines as the embodiment of energy dwelling within the main node, where all nodes share equal energy blessings.

But that is not all! We must not forget $X_{max}$ and $X_{min}$, the guiding lights that reveal the extremes of distance separating our beloved base sink and the sensor nodes. As the story unfolds, a magical event transpires upon the arrival of a push notification from a humble sensor. The network awakens, and a neighboring master node, carefully selected from the closest kin, assumes its noble duty.

Now, picture this: The base station, ever watchful, weaves a matrix of wisdom, casting it upon the winds to reach every sensor node within its reach. It is within this matrix that secrets reside, unveiling the intricate tapestry of our network’s essence, fostering seamless communication, and illuminating the path to informed decisions. Behold, the grand display of knowledge and connectivity, where *X*($Q_{a},BS)$ dances with w and g, the NN claims its zenith, and the energy of I−Remaning and I−Maximum intertwines in harmonious balance.

(5) X12K,…,XnKXabkX12K,…,Xn1K.

The network architecture incorporates a matrix that captures the interrelationships among nodes belonging to a particular tier, denoted as k. This matrix, denoted as XK ab, provides crucial information regarding the distances between each pair of nodes (a and b) and their corresponding NNs identified by the unique NN ID. By leveraging this matrix, individual sensors gain accurate insights into their specific NN and the distances to other sensors within the network. This knowledge empowers the sensors to optimize their transmission power levels based on the acquired distances, thereby enhancing overall energy efficiency in the system. This efficient utilization of the matrix facilitates seamless and reliable communication among the sensors while conserving valuable network resources.

#### 3.1.8. Neighbor Head Node Selection and Path Determination Phase

During the neighbor head node selection and path determination phase, the sender node SI considers a total of n neighboring nodes within its transmission range. The priority order of these nodes plays a crucial role in determining which node will govern the most efficiently and dependably. Once the most optimal network node has been identified, it is selected as the NHN among the remaining network nodes. The NHN is responsible for collecting data from nodes within its neighbors and transmitting it to the sink or other NHN. The NHN selection process is based on the residual energy of the node, its distance from the sender node, and its capacity to communicate with other nodes. The node with the greatest weight value is chosen as the NHN. Using the neighbor node cooperation mechanism described earlier, the chosen NHN then transmits the data to the destination node. Distance between nodes and the energy levels of each node are utilized to determine the routing path. The proposed NBEER protocol employs NNS and collaboration to determine the optimal data transmission routing path.

Sink nodes send hello messages to the network every 50 s during the neighbor head node selection and routing phase to detect any inactive nodes and calculate network parameters. When a destination is effectively reached, acknowledgements are broadcast to nearby sender nodes, eliminating the need for additional transmissions via nearby nodes. The sender node transmits messages to the neighbor node, which then identifies the payload and transmits it undetected to the sink node. When there are multiple NHNs along the route, the NHN is not responsible for initiating or generating a collaborative process when the sending node is a sink node with its subsequent steps, thereby reducing the residual energy after transmission. The eighth and ninth equations are used to exemplify this method.

(6) $$E_{re}(S_{i})>E_{re}\left(R_{i}\right)Then\;direct\;transfer$$

(7) $$Else\;E_{re}{(S}_{i})\leq E_{re}(R_{i})\;then\;relay\;\left(CHs\right)\;path$$

#### 3.1.9. Neighbor Head Node Methodology

The neighbor head node methodology considers the amplify and forward (AF) method, in which the neighbor head node (*Ri*) multiplies the acknowledgment signal from *Si*, from the beginning of transmission to the destination (*Di*), by a gain factor *G*, i.e., Y−rd=G(Y−sr). This method implies that the neighbor head node energy consumption is equivalent to that of the sending node during the first hop. Equations (8) and (9) can be used to express the amplifier parameter *G* in the presence of a channel state information (CSI) surge on the neighbor node.

(8) $$G=(|h_{SR}{|}^{2}+\frac{1}{y_{0}}{)}^{-1}$$

where $y_{o}=p_{s/N_{o}}$ is the common SNR of each link without fading: Y−rd=G(Y−sr).

(9) $$y_{RD}=h_{RD}Gy_{SR}+N_{RD}$$

The notation $h,n\in (S_{D,}S_{R,}R_{D})$ represents the fading channel magnitude.

#### 3.1.10. Integration Approach

The variable y_d represents the combined output signal at the destination node D in Equation (10). It is the sum of the instant signal-to-noise ratio (SNR) of the link between the sender node S and the receiver node D(y_SD) and the instant SNR of the link between the NHN R and the receiver node D(y_RD), respectively. Maximum Ratio Combining (MRC) is the hybrid combining method used here, which combines the two signals at the destination node to enhance the system’s efficiency.

(10) $$y_{d}=y_{SD}+y_{RD}$$

The pseudo code of the proposed work is given in Algorithm 1.
**Algorithm 1**: For the proposed NBEER routing protocol for UWSN1.import networking_ library2.import communication_ library3.Fi = forwarder nodes4.rmin = minimum radius5.St = sink node6.SJ = neighbor head node (NHN)7.rJ = advertisement packet8.Sn = all candidates9.p = routing path10.# Function to select neighbor node based on a specific criteria11.def select_ neighbor (nodes):12.Implement neighbor node selection logic here13.selected_ neighbor = None14.Select neighbor node based on desired criteria15.return selected_ neighbor16.Function to perform cooperative data transmission17.def transmit_ data (source, destination, data):18.Implement cooperative data transmission logic here19.Pass20.while (TTL > 0) and (Fi! = St):21.Fi. Sn = Ø22.Networking _ library. Transmit (Qi, broadcasting packet with neighbor head node rmin)23.for all SJ with δ (i, j) < rmin:
a.if cos (TTL) < 0:
i.communication _ library. Sleep (SJ)
b.else:c.St. Sn. Add (SJ)
24.Networking_ library. transmit (SJ, broadcasting packet with neighbor head node rJ)25.for all S k with δ (j, k) < rJ:26.if (εresk < ε resj):27.communication_ library. Sleep (S k)28.else:29.Fi.Sn. add (S k)30.Data transmission from NHN to sink31.for NHN in St. Sn:32.data = communication _library. Collect _ data (NHN)33.selected_ neighbor = select_ neighbor (Fi. Sn)34.transmit _ data (NHN, selected _neighbor, data)35.end if36.end for37.end if38.end for39.end while

The node parameter table lists the specifications of the simulation’s sensor nodes. This consists of the principal energy of normal nodes (22 joules), the transmission range of each node (250 m), and the quantity of NNS/relay nodes (20).

Existing Co-UWSN and CEER schemes are compared to the performance of the proposed scheme. In each one-thousandth simulation cycle, the data collection nodes are assigned at random. The network consists of 250 nodes randomly positioned in a 550 m × 450 m × 350 m field, as shown in Table 2, Table 3, Table 4, Table 5 and Table 6. Various parameters that are used in the simulations are shown, and twelve sinks are situated on the surface of the water.

## 4. Experiments

The Results and Discussion section of this research article presents a comprehensive analysis of the outcomes obtained from the proposed routing protocol, NBEER, in comparison to state-of-the-art protocols, namely Co-UWSN and CEER. This study focused on evaluating the performance of these protocols using key parameters, including end-to-end delay, total energy consumption, packet delivery ratio, alive nodes, and the number of packets received (NPR). By examining these metrics, we gain valuable insights into the effectiveness and efficiency of NBEER in relation to its counterparts. The subsequent discussion delves into the implications of the results, highlighting the strengths and weaknesses of each protocol and shedding light on potential avenues for further enhancements in underwater wireless sensor network (UWSN) routing protocols.

### 4.1. End-to-End Delay

**Figure 3:**
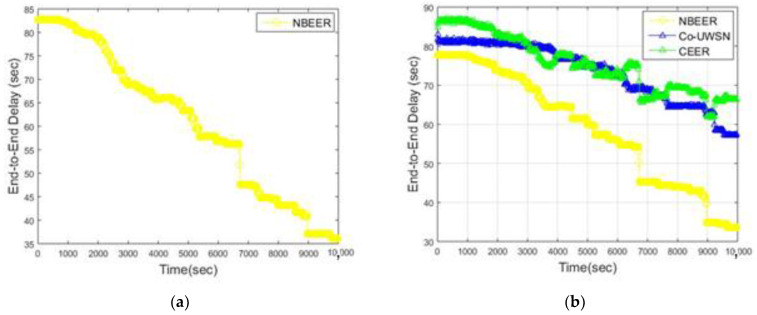
(**a**) End to End delay of the Proposed work (**b**) End-to-end delay of Present and Proposed Works vs. time (s).

Figure 3a presents the end-to-end delay of the proposed NBEER protocol, while Figure 3b showcases a comparison between NBEER and state-of-the-art routing protocols such as Co-UWSN and CEER. In this analysis, the end-to-end delays of NBEER, Co-UWSN, and CEER were evaluated and compared using Table 7. The study incorporated simulations of various technologies not considered in the analysis, and their results were integrated into the findings. From the observations in Figure 3a,b, it is evident that NBEER exhibits lower end-to-end latency compared to competing technologies. This advantage can be attributed to the shorter minimum forwarding distance between nodes in both dense and sparse conditions.

On the other hand, Co-UWSN and CEER experience increased end-to-end delays in the final round due to extended data transmission distances. Network stability is achieved after approximately 4000 rounds as the network sparsity gradually increases, leading to data transmission over the shortest feasible distance. Co-UWSN and CEER, through threshold modifications and weighting functions, facilitate network load balancing, resulting in shorter end-to-end delays compared to NBEER. These modifications also contribute to the transmission of more reliable packets, thereby minimizing retransmissions. This characteristic is particularly advantageous for collaborative schemes like Co-UWSN and CEER, which are gaining popularity in the field.

**Figure 4:**
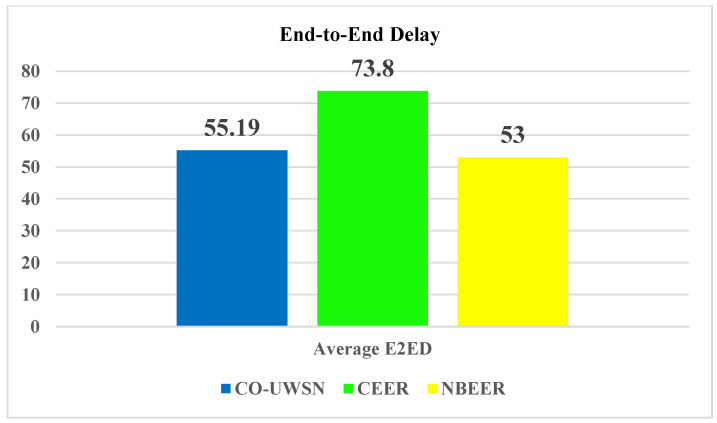
Average values of end-to-end delay.

Figure 4 shows the average end-to-end delay values, highlighting NBEER’s advantage over Co-UWSN and CEER, with NBEER exhibiting an average end-to-end delay of 53, outperforming Co-UWSN (55.19) and CEER (73.8). The successful implementation of neighbor node cooperation and direct data transfer contribute to NBEER’s exceptional performance in comparison to other state-of-the-art protocols.

### 4.2. Total Energy Consumption

**Figure 5:**
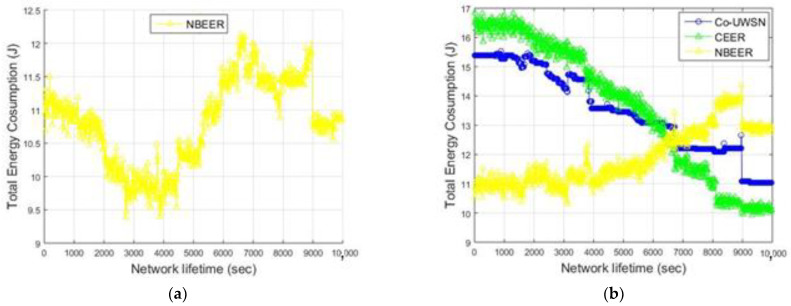
(**a**) TEC of Proposed Work (**b**) Total energy consumption of all protocols vs. network lifetime.

Figure 5a,b compare NBEER’s total energy consumption to that of other energy-efficient schemes. By collaborating with neighboring nodes, our scheme optimizes data forwarding and load balancing, resulting in more efficient energy consumption by the system’s sensors. Additionally, weight implementation efficiency and data transmission efficiency contribute to overall energy savings. Co-UWSN and CEER, on the other hand, consume more energy due to their emphasis on time-sensitive application requirements and reliance on collaboration and depth differences between data forwarders. This frequently results in the consistent selection of high-energy nodes, reducing the likelihood of collaborative routing at any source node, and resulting in an increase in energy consumption.

**Figure 6:**
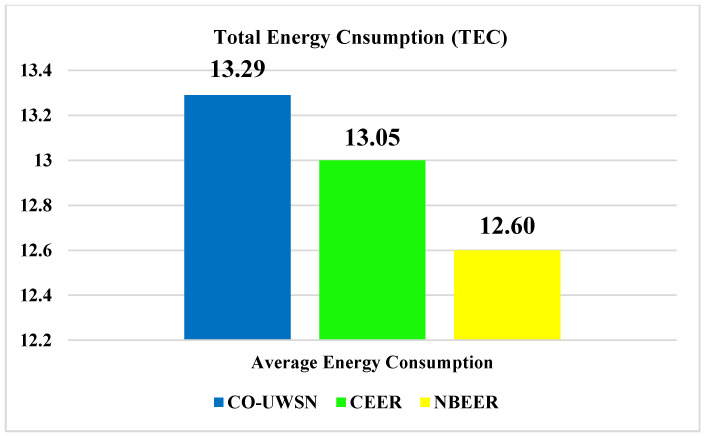
Average values of total energy consumption.

Figure 5a displays the total energy consumption of the proposed NBEER protocol, while Figure 5b compares NBEER’s energy consumption with that of Co-UWSN and CEER. NBEER exhibits lower energy consumption due to neighbor node cooperation and the selection of an NHN for data transmission. By utilizing NHNs, NBEER minimizes the energy usage of the NHN only, resulting in reduced overall energy consumption compared to when all nodes are involved. The strategic NHN selection ensures efficient and energy-saving data transfer, making NBEER a highly energy-efficient protocol compared to Co-UWSN and CEER. Figure 6 shows a comparison of the average total energy consumption: Co-UWSN obtained a value of 13.39, CEER 12.01, and NBEER 10.33.

As shown in Figure 5b and Table 8, the energy consumption of the NBEER protocol increased as the network lifetime progressed. This increase can be attributed to the following factors.

Node cooperation and neighbor node selection: In the initial stages of network operation, when the network lifetime is relatively low, nodes have ample energy reserves. This allows for easier cooperation and the selection of neighbor head nodes (NHNs). However, as the network operates for a longer duration, the overall energy consumption increases due to the accelerated selection of NHNs. This is because the network becomes fully operational, and the selected NHNs actively send data to the sink nodes and the base station. The average values of each protocol has been given in Figure 6.

### 4.3. Packet Delivery Ratio

**Figure 7:**
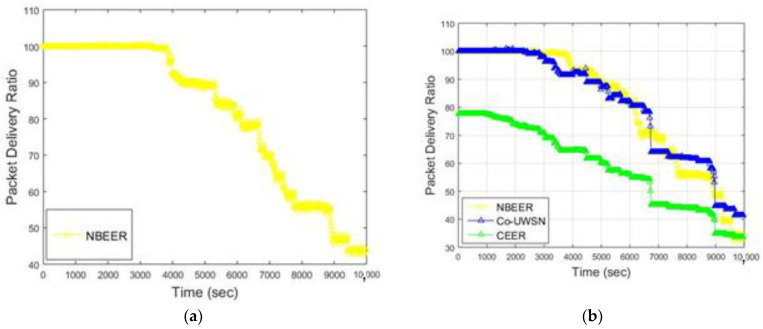
(**a**) Packet Delivery Ratio of NBEER (**b**) Packet delivery ratio of all protocols vs. time (s).

Figure 7a presents the PDR of the proposed NBEER protocol, while Figure 7b and Table 9 provide a comparison of NBEER’s PDR with that of Co-UWSN and CEER. Notably, NBEER outperforms the other protocols with significantly higher PDR values. This superiority is attributed to neighbor node cooperation and the selection of an NHN for data transfer. By leveraging NHNs, NBEER effectively minimizes packet loss and achieves a remarkable PDR.

**Figure 8:**
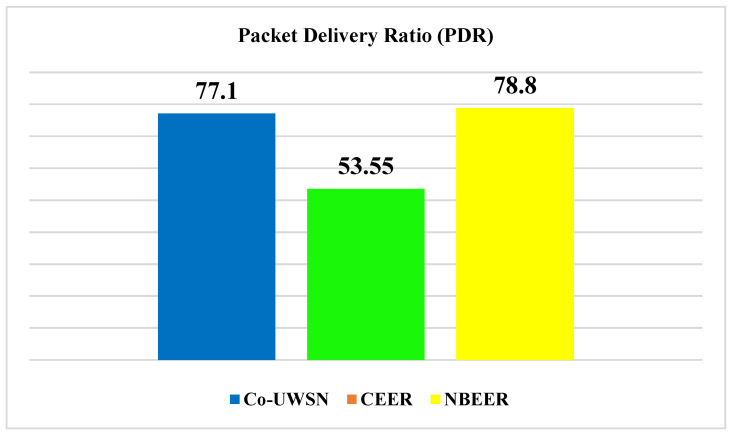
Average values of packet delivery ratio.

Furthermore, Figure 8 highlights the average PDR values of the protocols. Co-UWSN demonstrates a PDR of 42.47 and CEER shows a PDR of 53.55, while NBEER excels with an impressive average PDR of 81.1. These results reaffirm the enhanced performance and reliability of NBEER, showcasing its ability to maintain high packet delivery rates in underwater wireless sensor networks.

The proposed protocol uses the neighboring-based method, in which each node tries to avoid any loss and thus estimate the highest packet delivery ratio. The same procedure is implemented with the proposed NBEER protocol, which shows a greater ratio in comparison with Co-UWSN. In Co-UWSN, only the cooperation scheme has been followed previously. But, in the proposed NBEER protocol, there is cooperation, and neighboring-based node implementation takes place. This is the key factor allowing the proposed protocol, NBEER, to achieve a greater packet delivery ratio compared to Co-UWSN and the others as well.

### 4.4. Number of Alive Nodes

**Figure 9:**
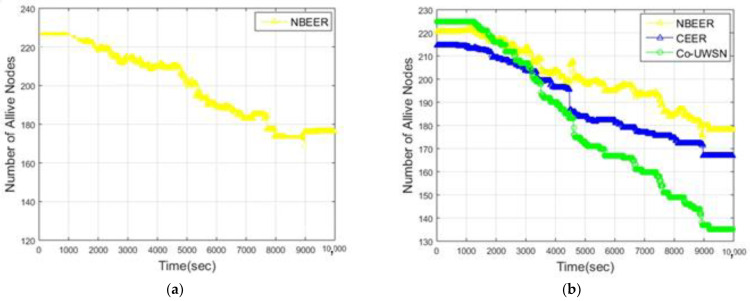
(**a**) Alive nodes analysis of NBEER (**b**) Alive Node of all protocols versus network lifetime (s).

Figure 9a displays the number of alive nodes in the proposed NBEER protocol, while Figure 9b and Table 10 compare the number of alive nodes between NBEER, Co-UWSN, and CEER. NBEER demonstrates a higher number of alive nodes, indicating its effectiveness in maintaining network connectivity.

In NBEER, the higher number of alive nodes can be attributed to the minimal energy consumption and efficient cooperation between nodes, enabling easy data transfer to the sink node. Conversely, in CEER and Co-UWSN, clustering with cooperation leads to increased energy consumption. This is primarily because these techniques involve a multi-step process where clusters are first formed, cluster heads are then selected based on maximum energy, and subsequent cooperation among cluster heads takes place. Such lengthy procedures result in higher energy consumption and depletion of node energy.

**Figure 10:**
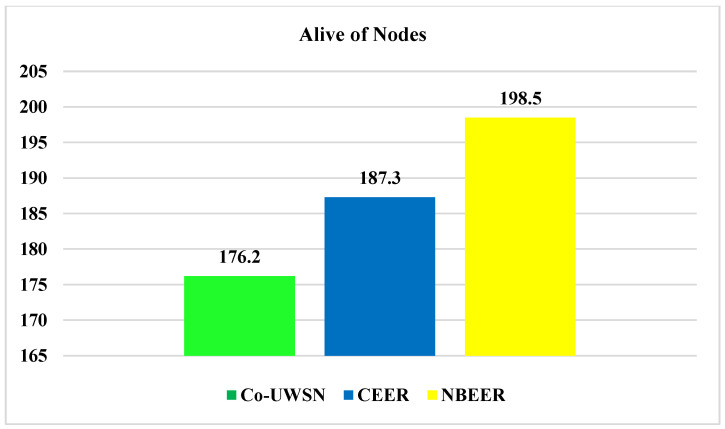
Comparison of average values of alive nodes of NBEER with other state-of-the-art protocols.

In our proposed technique, we address these issues by introducing a more streamlined approach. Instead of forming clusters and selecting cluster heads, nodes directly choose neighboring nodes as head nodes. Data are then transferred from the nodes to the selected head nodes, and the head nodes transmit the data directly to the sink node. This approach significantly reduces energy consumption as only a single node, the NHN (neighboring head node), is responsible for transmitting data. As a result, the remaining nodes conserve their energy and remain unaffected. Figure 10 presents a comparison of the average values, highlighting NBEER’s superiority in terms of the number of alive nodes. This underscores the efficacy of neighbor node cooperation and NHN selection in NBEER, contributing to enhanced network longevity and connectivity. In summary, NBEER’s neighbor node cooperation and NHN selection led to a higher number of alive nodes, optimizing energy utilization and bolstering network vitality. The comparison in Figure 10 reinforces NBEER’s advantage in sustaining a robust underwater wireless sensor network infrastructure.

- Iterations of neighboring and Dispersion of Neighbor Head Nodes

The algorithmic overhead is influenced by the number of iterations involved in the compilation process. By reducing the iterations per round, the build time and the number of transmitted packets can be minimized. The relationship between the initial value 0 and the neighboring node (NN) numbers, denoted as “n”, becomes apparent when considering two different values, denoted as “w” and “y”. When both “w” and “y” are set to 0, the minimum number of NNs required to increase the CR (communication reliability) is achieved. As the NN count increases, the minimization of “w” and “y” values in CR becomes crucial. In our proposed method, the values of “w” and “y” are both set to 1, and a CR value of 60 is assigned. The battery life of the nodes within the network is dependent on the total number of nodes present.

### 4.5. Number of Packets Received (NPR)

**Figure 11:**
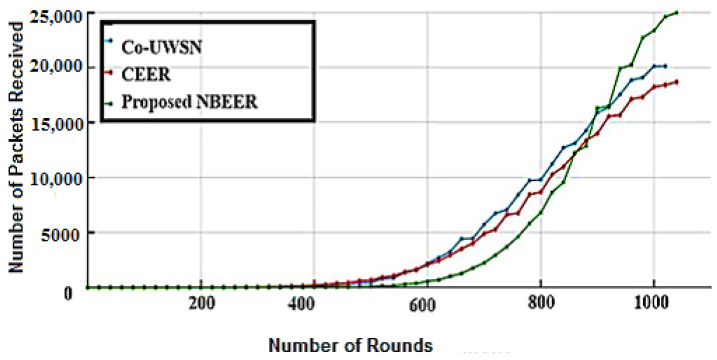
Total received packets analysis.

This section presents a comparative analysis of the number of packets received (NPR) when employing the neighbor node analysis technique against alternative methodologies across multiple iterations illustrated in Figure 11. Empirical findings reveal that the NBEER design strategy yielded suboptimal outcomes in comparison to the conventional approach. Notably, NBEER’s approach marginally outperformed Co-UWSN and CEER in terms of NPR. However, the NPR improvement achieved by NBEER through iterative rounding was only modestly superior to the initial NPR. While the CEER method aimed to achieve a reasonable NPR, the NBEER method demonstrated the ability to attain a higher NPR compared to other approaches.

In accordance with Figure 10, the NBEER methodology emerges as a commendable strategy for optimizing energy efficiency and prolonging the lifespan within the framework of an UWSN. The comprehensive outcomes of the experiment reveal that the CEER technique exhibited unsatisfactory performance across all NPR assessment criteria [15]. Co-UWSN performed better than CEER, and ultimately, our proposed approach outperformed all alternative methodologies, yielding superior outcomes.

## 5. Conclusions

This study proposes the NBEER scheme to extend a network’s lifetime while reducing its energy consumption. Collaborative schemes and clustering mechanisms prolong the lifecycle of a network and maximize PDR, E2ED, total energy consumption, stability period, and number of packets received, thereby reducing the network’s total energy consumption. This is particularly important and advantageous when dealing with time- and delay-sensitive applications. However, when communicating over a single path, channel quality variations may cause routing and path selection issues. Collaboration-free transmission protocols use channel estimation to improve the quality of packets received by the destination node. To forward packets to other NHNs efficiently, it is necessary to select an NHN scheme that takes into consideration the burstiness of the link and the distance between nearby NHNs. The ultimate destination is the BS. By alternating the depth thresholds, the number of applicable NHNs increases, thereby mitigating the loss of critical data in time-sensitive applications. Single-hop and multi-hop transmission methods have been utilized to mitigate conflicts in situations where path loss is a concern and to maximize the network’s lifetime. Not only do optimal weight calculation and collaboration feature aid in balancing a network’s load, but they can also substantially extend the network’s period of stability.

Our study contributes to the development of energy-efficient routing techniques in UWSNs, offering potential for more sustainable and effective underwater communication networks. NBEER’s neighbor-based approach and cooperative strategies address critical challenges, paving the way for improved performance and longevity in UWSNs.

In the future, it may be possible to develop a mathematical model that will enable us to reduce our energy consumption even further. New energy consumption techniques, like converting a power supply to solar energy using multiple parameters, such as depth, energy, data traffic, and the number of neighboring nodes, are utilized to optimize the UWSN data forwarding strategy.

## Figures and Tables

**Table 1:** Compilation of newly released approaches.

Ref.	Year	Utilized Technique	Benefits	Limitations
[22]	2022	A blend of approaches is employed to select forwarder nodes for data transmission, encompassing both single and multipath routing schemes. Various factors, i.e., minor bit error rate (BER), minimum distance to the sink node, and highest residual energy, are considered during the selection process.	Enhanced energy efficiency and dependable packet delivery	Increased latency caused by node collaboration
[23]	2021	Multipath routing techniques are employed for efficient data transfer among the nodes.	Improved the PDR (packet delivery ratio)	Increases the latency and decreases the reliability
[24]	2020	The selection of the best forwarder nodes is based on the weight function, and the scheme incorporates the use of the Maximum ratio combining (MRC) technique for improved performance.	Improved the network reliability	More energy consumption
[25]	2019	Data transmission utilizes the Manhattan and RSSI techniques for enhanced efficiency.	Improved transmission latency and energy construction	More latency due to hierarchical structure
[26]	2019	Both the collaborative and independent approaches employed the positional data of nodes, considering both proximity and the movement of sinks, to facilitate the propagation of information.	Reduces latency and enhances throughput	Less reliability and high propagation delay
[40]	2018	The non-cooperative routing scheme employes a designated area of interest.	Improved network lifetime, reliability, and throughput	Data transmission limited to a single random node occurrence
[41,42,43]	2017	Clustering-based cooperative techniques; nodes cooperate within clusters to send and receive data.	Consumes less energy	A lower packet delivery ratio due to node cooperation

**Table 2:** Simulation parameters.

Parameter	Value
Total Deployed NNS Schemes	10
Neighbor Node Heads	12
Primary Energy of Normal Nodes	22 joules
Transmission Range	250 m
Performance Parameter	Packet Delivery Ratio Alive vs. Dead Nodes End-to-End Delay Energy Consumption Number of Packets Received
Channel and Frequency Type	Acoustic Channel VLF radio waves (3–30 kHz)
Frequency Range	2.412 GHz to 2.472 GHz
Number of NNS/Relay Nodes	20
Packet Size	512 bytes

**Table 3:** General simulation parameters.

Parameter	Value
Simulator	MATLAB
Network Area	550 m × 450 m × 350 m
Number of Deployed Nodes	250
Total Deployed NNS Schemes	10
Simulation Time	1000 s

**Table 4:** Node parameters.

Parameter	Value
Primary Energy of Normal Nodes	22 joules
Transmission Range	250 m
Number of NNS/Relay Nodes	20

**Table 5:** Communication parameters.

Parameter	Value
Frequency Range	The frequency range of the channel spans from 2.412 GHz to 2.472 GHz.
Packet Size	512 bytes

**Table 6:** Underwater acoustic communication parameters.

Parameter	Value
Acoustic Propagation Model	Bellhop or Ray Tracing
Sound Speed Profile	Empirical or Custom
Transmission Frequency	10 kHz to 50 kHz
Transmission Power	160 dB re 1 μPa @ 1 m

**Table 7:** End-to-end delay after equal intervals of time.

	E2ED	E2ED	E2ED	E2ED	E2ED	E2D	E2ED	E2ED	E2ED	E2ED	Avg
**Protocol**	**After**	**After**	**After**	**after**	**After**	**after**	**After**	**After**	**After**	**After**	**E2ED**
	**1000 s**	**2000 s**	**3000 s**	**4000 s**	**5000 s**	**6000 s**	**7000 s**	**8000 s**	**9000 s**	**10,000 s**	
**Co-UWSN**	**84.03**	**80.01**	**73.02**	**60.01**	**53.03**	**51.01**	**46.01**	**43.03**	**34.03**	**34.01**	**55.19**
**CEER**	**86**	**82**	**79**	**76**	**75**	**72**	**68**	**70**	**62**	**68**	**73.8**
**NBEER**	**78**	**74**	**68**	**65**	**60**	**57**	**45**	**44**	**42**	**25**	**53.03**

**Table 8:** Total energy consumption after equal intervals of time.

	TEC	TEC	TEC	TEC	TEC	TEC	TEC	TEC	TEC	TEC	Avg
**Protocol**	**after**	**after**	**After**	**after**	**After**	**after**	**after**	**After**	**After**	**After**	**TEC**
	**1000 s**	**2000 s**	**3000 s**	**4000 s**	**5000 s**	**6000 s**	**7000 s**	**8000 s**	**9000 s**	**10,000 s**	
**Co-UWSN**	**17.35**	**15.0**	**14.19**	**12.98**	**12.68**	**12.0**	**11.48**	**10.01**	**9.95**	**9.79**	**13.29**
**CEER**	**16.93**	**15.62**	**11.26**	**9.54**	**8.31**	**7.113**	**6.56**	**6.145**	**5.93**	**4.93**	**13.15**
**NBEER**	**16**	**16.2**	**16.3**	**13**	**12.0**	**11.9**	**10.8**	**9.07**	**8.15**	**8.01**	**12.60**

**Table 9:** Packet delivery ratio at equally spaced time intervals.

	PDR	PDR	PDR	PDR	PDR	PDR	PDR	PDR	PDR	PDR	Avg
**Protocol**	**After**	**After**	**After**	**After**	**After**	**After**	**after**	**after**	**After**	**After**	**PDR**
	**1000 s**	**2000 s**	**3000 s**	**4000 s**	**5000 s**	**6000 s**	**7000 s**	**8000 s**	**9000 s**	**10,000 s**	
**Co-UWSN**	**100**	**100**	**98**	**90**	**88**	**80**	**65**	**61**	**57**	**42**	77.1
**CEER**	**99.51**	**95.51**	**78.95**	**65.22**	**48.78**	**41.88**	**38.94**	**24.21**	**24.21**	**22.34**	53.55
**NBEER**	**100**	**100**	**100**	**93**	**91**	**82**	**72**	**58**	**56**	**36**	78.8

**Table 10:** The number of alive nodes and average alive nodes after 10,000 s.

	First Node Dies at s	Alive Nodes	Alive Nodes	Alive Nodes	Alive Nodes	Alive Nodes	Alive Nodes	Alive Nodes	Alive Nodes	Alive Nodes	Alive Nodes	Average Alive Nodes
**Protocol**		**After**	**After**	**After**	**After**	**After**	**After**	**After**	**After**	**After**	**After**	
		**1000 s**	**2000 s**	**3000 s**	**4000 s**	**5000 s**	**6000 s**	**7000 s**	**8000 s**	**9000 s**	**10,000 s**	
**Co-UWSN**	**551**	**225**	**217**	**207**	**191**	**170**	**168**	**160**	**150**	**139**	**135**	**176.2**
**CEER**	**529**	**215**	**209**	**204**	**198**	**182**	**180**	**178**	**172**	**168**	**167**	**187.3**
**NBEER**	**633**	**220**	**218**	**212**	**203**	**198**	**195**	**192**	**184**	**181**	**180**	**198.3**

## Data Availability

Not applicable.
